# Health-related quality of life in multiple sclerosis: temperament outweighs EDSS

**DOI:** 10.1186/s12888-018-1719-6

**Published:** 2018-05-23

**Authors:** S. Salhofer-Polanyi, F. Friedrich, S. Löffler, P. S. Rommer, A. Gleiss, R. Engelmaier, F. Leutmezer, B. Vyssoki

**Affiliations:** 1 Department of Neurology, Medical University of Vienna, Währingergürtel 18-20, 1090 Vienna, Austria; 2 Department of Psychiatry and Psychotherapy Division of Social Psychiatry, University of Vienna, Vienna, Austria; 3 Center for Medical Statistics, Informatics, and Intelligent Systems of the Medical, University of Vienna, Vienna, Austria

**Keywords:** Temperament, Multiple sclerosis, Health-related quality of life, TEMPS, MusiQol

## Abstract

**Background:**

The influence of personality on health-related quality of life in patients with multiple sclerosis has been the focus of previous studies showing that introversion and neuroticism were related with reduced health related quality of life. However, no data exist on the impact of temperament on quality of life in this patient group.

**Methods:**

Between April 2014 and March 2016 139 multiple sclerosis patients were recruited from a specialized outpatient clinic of the general hospital of Vienna. Health-related quality of life was measured by “The Multiple Sclerosis International Quality of Life Questionnaire (MusiQol)”, temperament by “Temperament Evaluation of Memphis, Pisa, Paris, and San Diego Questionnaire – Münster version” (briefTEMPS-M), and disability by the “Expanded disability status scale”. All patients underwent a diagnostic psychiatric semi-structured interview (MINI).

**Results:**

Known predictors (like disease duration, EDSS, psychiatric co-morbidities, immunomodulatory treatments) explain the proportion of variation in the outcome of MusiQol global index score in 30.9% in multi-variable linear regression analysis. It increased respectively to 40.3, 42.5, and 45.8% if adding the depressive, cyclothymic, or hyperthymic temperament to the list of variables. An increase of depressive and cyclothymic temperament scores significantly reduced global index score of MusiQol (*p* = 0.005, *p* = 0.002, respectively), while the hyperthymic temperament significantly raised it (*p* < 0.001).

**Conclusion:**

In MS patients, the depressive and cyclothymic temperament predict a lower and hyperthymic temperament an increased health-related quality of life, independent of current disability status, immunomodulatory treatments, and affective co-morbidities.

**Electronic supplementary material:**

The online version of this article (10.1186/s12888-018-1719-6) contains supplementary material, which is available to authorized users.

## Background

Multiple sclerosis (MS) is a chronic demyelinating and neurodegenerative disorder of the central nervous system. It is characterized by clinical exacerbations alternating with episodes of complete or relative well-being [1]. MS challenges patients to deal with its typically unpredictable clinical course and MS is known to have negative effects on quality of life and mental health. A high rate of psychiatric co-morbidities like anxiety and mood disorders can be found [2–4]. Further, in previous studies personality accentuations have been reported to be common in MS, which play a role in the disease course and influence quality of life [5–7]. Zarbo et al., for example, recently could show, that introversion and neuroticism are associated with lower health-related quality of life in MS and concluded that health related quality of life is largely influenced by these personality traits [8].

However, to our best knowledge, no studies were published, which focused on the impact of temperament on health related quality of life in MS, which can be seen as an individual’s core of behavior and affectivity, on which personality traits are acquired on top. While personality is influenced by personal experience, temperament is defined as an emotional, inherited, and temporally stable domain of personality [9, 10]. Based on the research of Emil Kraepelin [8], Akiskal [10] described five temperament types (i.e. depressive, cyclothymic, hyperthymic, irritable, and anxious temperament) [10]. These temperament types represent healthy emotional reactivity patterns and stretch to the earliest subclinical presentations of affective disorders, always depending on whether a temperament is expressed in a predominant form [9]. Only the study by Özkan et al. using TEMPS-A [10], investigated the influence of temperament, according to Akiskals concept, in MS previously. However, focusing on mood disorders, quality of life was not assessed in this study. They found that MS patients scored higher in the depressive, cyclothymic, irritable, and anxious domains than the control group [11]. Studies using “Cloninger Temperament and Character Inventory” for temperament evaluation in MS patients found elevated levels of harm avoidance and lower levels of reward dependence and persistence as compared to healthy controls [6, 12]. The quality of life was not evaluated in these studies.

The aim of our study was therefore to evaluate the impact of different temperament types as defined by Akiskal et al. [9] on health-related quality of life in MS patients.

## Methods

Between April 2014 and March 2016 patients were recruited from the outpatient MS clinic of the Medical University of Vienna and gave written informed consent to participate. Inclusion criteria were a diagnosis of multiple sclerosis according to the revised McDonalds criteria [13] and a minimum age of 18 years. Secondary progressive MS was defined as continuous worsening of EDSS in the absence of a relapse over a period of 12 months in patients formerly suffering from relapsing remitting MS. Patients suffering from clinical relevant psychiatric disorders, other than affective disorders, within 6 months from the inclusion time point were excluded.

After screening of 151 MS patients and exclusion of 12 patients due to psychiatric comorbidities, a final number of 139 MS patients were included.

The study was approved by the ethics committee of the Medical University of Vienna (EK1715/2013).

### Evaluation of health-related quality of life

Health-related quality of life was evaluated by “The Multiple Sclerosis International Quality of Life questionnaire” (MusiQol) [14]: a multidimensional, self-administered, patient-focussed questionnaire that refers to the last four weeks and consists of thirty-one items, describing nine dimensions of health-related quality of life (i.e. activities of daily living, psychological wellbeing, symptoms, relationship with friends, relationships with family, sentimental and sexual life, coping, rejection and relationship with healthcare system). All sub-scores and the global index score are linearly transformed with a maximum sum score of 100 with higher scores indicating a better health related quality of life. According to the manual’s procedure, calculation of global index score is only recommended for patients for whom all of nine dimensions could be calculated (a dimension’s value is set to missing if at least 50% of the items within the dimension are missing) [14].

### Evaluation of temperament type

Temperament was evaluated by brief-TEMPS-M (Temperament Evaluation of Memphis, Pisa, Paris, and San Diego questionnaire – Münster version) [15]: a short, self-administered questionnaire consisting of thirty-five items with a 5-point anchored Likert-type scale, assessing five types of temperaments as suggested by Akiskal et al. [9]: the depressive, hyperthymic, cyclothymic, irritable, and anxious temperament.

### Evaluation of psychiatric disorders

The “Mini International Neuropsychiatric Interview” (MINI) [16] is a semi-structured interview and was used to diagnose mental disorders according to the DSM-IV standards [17].

### Evaluation of disability

The “Expanded disability status scale” (EDSS) [18] was used to assess disability in MS patients. The EDSS is an ordinal clinical rating scale, ranging from 0 to 10 with higher scores indicating more severe disability. Scoring is based on clinical neurological examination.

### Statistical analysis

Categorical variables are described as counts and percentages. Continuous variables are described as medians and ranges due to the asymmetric distribution of most variables. The potential effect of each of five temperament scores on the MusiQoL global index score was investigated in five separate multi-variable linear regression models to correct for the influence of known predictors (disease duration, EDSS, psychiatric co-morbidities, immunomodulatory treatments). All temperament scores except hyperthymic exhibited a clearly right-skewed distribution such that these scores were transformed for use in the regression models using a logarithmic transformation with base 1.2. Thus, regression coefficients (reported with 95% confidence intervals) quantify the effect of a 20% temperament score increase on the global index score, while for hyperthymic temperament it quantifies the effect of a temperament score increase by one score point. The *p*-values for the five temperament scores are adjusted for multiple testing using the method of Bonferroni-Holm. For each model, we also report the R^2^ to quantify the proportion of variation in the outcome (global index score) that is explained by the variables in the respective model.

The strict application of the MusiQoL manual’s procedure assigns a missing value in the global index score if at least one of the nine dimensions is missing. This approach resulted in missing global score index values for 58 of 139 patients. In a sensitivity analysis, we therefore calculated the global index score not only for those patients with non-missing values for all nine dimensions, but for the 132 patients with at least five non-missing dimensions.

Each of MusiQoL dimensions 1 to 3 was investigated in the same manner as described above for the global index score. Dimensions 4 to 9, however, showed high percentages (30 to 60%) of patients obtaining full score precluding an investigation by linear regression models. Instead, we dichotomized each dimension to ‘full score’ versus ‘below full score’. For each dimension, the dichotomized score was investigated in a separate logistic regression model with the same four known predictors as above and each temperament score. The effect of the latter is quantified by an odds ratio. For each dimension the *p*-values corresponding to the five temperament scores are again adjusted for multiple testing using the method of Bonferroni-Holm. The importance of temperament scores is quantified using proportions of explained variation [19]. Note that these are known to be considerably lower in general than R^2^ values in linear regression.

The reported *p*-values are the results of two-sided tests. *P*-values ≤0.05 were considered to be statistically significant. Since the global index score was the primary outcome and the nine dimensions were investigated in an exploratory manner, no correction for testing nine dimensions has been performed; p-values and confidence intervals have to be interpreted accordingly. All computations were carried out using SAS software Version 9.4 (SAS Institute Inc., Cary, NC, USA, 2012).

## Results

**Fig. 1:**
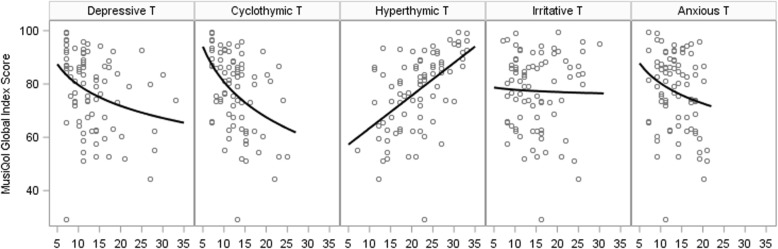
Scatter plot and simple regression line depicting the association of MusiQol global index score with each temperament score. Footnote: T: temperament. Regression lines for temperaments (exluding hyperthemic) are based on log of temperament score but shown on original scale

Demographic characteristics of included patients are shown in Table 1. Median scores of the brief-TEMPS-M were 11 (range 7–33), 11 (range 7–30), 22 (range 7–35), 12 (range 7–30), and 13 (range 7–27) in the depressive, cyclothymic, hyperthymic, irritable, and anxious temperament, respectively (Fig. 1).

**Table 1:** Demographic characteristics

Parameter	MS
	*n* = 139
Median age, years (range)	40 (19–72)
Female (%)	70.5%
Median EDSS (range)	1.5 (0–8)
Annualized relapse rate (ARR)	0.58
Immunomodulatory treatment	69%
Median disease duration in months (range)	108 (1–492)
Disease course	
Relapsing remitting	76.3%
Secondary progressive	20.1%
Primary progressive	3.6%
Family Status	
Married	42.4%
Divorced	9.3%
Single	47.5%
Widowed	0.7%
Employment status	
Full-time employment	38.1%
Part-time employment	16.5%
Student	7.2%
Housewife	2.2%
Unemployed	36.0%

Calculation of global index score strictly according to the MusiQol’s manual was done in 81 patients, showing a median of 81.1 (range 29.3–88.4). The proportion of variation in the outcome of MusiQol global index score that was explained by known predictors (disease duration, EDSS, psychiatric co-morbidities, immunomodulatory treatments) amounts to 30.9% in multi-variable linear regression analysis. This percentage is increased to 40.3, 42.5, or 45.8% if depressive, cyclothymic, or hyperthymic temperament was added to the list of variables. Irritative and anxious temperament had no statistically significant influence. An increase of depressive and cyclothymic temperament scores significantly reduced global index score (adj. *p* = 0.005, *p* = 0.002, respectively), while hyperthymic temperament significantly raised it (*p* < 0.001). A detailed listing of these results is given in Table 2. Figure 1 depicts the association of global index score with each temperament score. See Additional  file 1: Table S1 for a sensitivity analysis covering 132 patients.

**Table 2:** Linear regression analysis is showing the effect of temperament types on MusiQol Global Index Score (*N* = 81)

	R^2^	effect (CI)	*p*-value	Adj. *p*-value
Global Index Score	0.309		–	–
Depressive T	0.403	−2.2 (−3.5; −0.9)	0.001	0.005
Cyclothymic T	0.425	−3.0 (−4.5;-1.4)	< 0.001	0.002
Hyperthymic T	0.458	1.0 (0.6;1.4)	< 0.001	< 0.001
Irritative T	0.310	−0.3 (−1.7;1.2)	0.717	1.0
Anxious T	0.319	−1.0 (−2.9;0.9)	0.293	0.880

**e**ffect: regression coefficient quantifying the effect of a 20% increase in the respective temperament score (except hyperthymic temperament: quantifying the effect of a unit increase in the temperament score); CI: 95% confidence interval; adj. *p*-value: adjusted for testing five temperaments (Bonferroni-Holm method). T: Temperament

We also analyzed each of the nine dimensions of MusiQol separately.

An increase of cyclothymic and anxious temperament score by 20% reduced the score of “activities of daily living” on average by − 2.8 score points (95% CI -4.6 to − 1.1, *p* = 0.002) and − 2,6 (95% CI -4.5 to − 0.7, *p* = 0.008), respectively. After adjusting for testing five temperaments, this was still statistically significant (*p* = 0.008, *p* = 0.0032, respectively). The score of “psychosocial wellbeing” was statistically significantly increased by hyperthymic temperament (*p* < 0.001) and decreased by depressive (*p* < 0.001), cyclothymic (*p* < 0.001), and anxious (*p* < 0.001) temperament. Table 3 gives detailed information on these results.

**Table 3:** Linear regression analysis is showing the effect of temperament types on MusiQol Dimensions 1–3

	N	R^2^	effect (CI)	p	Adj. p
Activities of daily living	132	0.52	–	–	–
Depressive T		0.53	−0.7 (−2.2; 0.9)	0.394	1.0
Cyclothymic T		0.56	−2.8 (−4.6; −1.1)	0.002	0.008
Hyperthymic T		0.53	0.2 (−0.3; 0.8)	0.377	1.0
Irritative T		0.52	0.4 (−1.3; 2.1)	0.663	1.0
Anxious T		0.55	−2.6 (−4.5; −0.7)	0.008	0.032
Psychosocial Wellbeing	133	0.29			
Depressive T		0.41	−3.6 (−5.0; −2.2)	< 0.001	< 0.001
Cyclothymic T		0.42	−4.3 (−5.9; −2.7)	< 0.001	< 0.001
Hyperthymic T		0.38	1.1 (0.6; 1.6)	< 0.001	< 0.001
Irritative T		0.29	−0.3 (−2.0; 1.4)	0.703	0.703
Anxious T		0.40	−4.3 (−6.1; −2.5)	< 0.001	< 0.001
Symptoms	134	0.19			
Depressive T		0.20	−1.2 (−2.8; 0.4)	0.141	0.281
Cyclothymic T		0.29	−3.9 (−5.6; −2.1)	< 0.001	< 0.001
Hyperthymic T		0.24	0.8 (0.3; 1.4)	0.003	0.013
IrritativeT		0.19	0.6 (−1.2; 2.4)	0.490	0.490
Anxious T		0.21	−2.0 (−4.0; 0.0)	0.051	0.152

effect: regression coefficient quantifying the effect of a 20% increase in the respective temperament score (except hyperthymic temperament: quantifying the effect of a unit increase in the temperament score); CI: 95% confidence interval; adj. p-value: adjusted for testing five temperaments (Bonferroni-Holm method). T: Temperamen

Logistic regression analysis was performed for the remaining MusiQol dimensions. The proportion of variation in the outcome of “relationship with family”, “sentimental and sexual life”, “coping”, “rejection”, and “relationship with health care system”, that is explained by known predictors (EDSS, disease duration, psychiatric co-morbidities, MS-specific medication), amounts to 5.9, 1.9, 5.2, 10.4, and 13.6%, respectively, and was increased to 10.7, 13.6, 10.7, 17.2, and 16.4%, respectively, after adding hyperthymic temperament to the list of variables. A unit increase in the hyperthymic temperament score reduced the odds of obtaining a full score in “sentimental and sexual life” and “rejection” by 12% (OR = 0.88, adj. *p* = 0.001) and 11% (OR = 0.89, adj. *p* = 0.014), respectively. An increase of depressive and cyclothymic temperament score by 20% increased the odds of obtaining a full score in “rejection” by 50% (OR = 1.50, adj. *p* = 0.005) and 42% (OR = 1.42, adj. *p* = 0.031), respectively. An increase of depressive and cyclothymic temperament scores also significantly increased the odds of obtaining a full score in “relationship with health care system” (adj. *p* = 0.001 and 0.004, respectively). See Additional file 2: Table S2 for more results.

## Discussion

To our best knowledge, this is the first study investigating the impact of affective temperament types on health-related quality of life in MS-patients. Analyzing data of 139 MS-patients, we found that the depressive and cyclothymic temperament predicted a lower and the hyperthymic temperament an increased health-related quality of life in MS patients, independent of current disability status. Accordingly, dealing with physical disability based on predetermined temperamental attributes rather than disability itself seems to affect HRQol in MS. Besides, the statistically significant effect of temperament types on HRQoL was also independent of co-morbid mood disorders and immunomodulatory treatments, both factors known to influence HRQoL of MS patients [18]. Depression and fatigue were independently associated with reduced HRQoL in another study [20] and the benefit of immunomodulation on HRQoL was most prominent in MS patients suffering from marked fatigue [21].

The high impact of affective temperaments on health-related quality of life highlights their alleged role as an individual’s core of behavior and affectivity and as earliest subclinical phenotypes of affective disorders [22]. This especially applies to the depressive and cyclothymic temperament, when considering that depressive mood ranks among the most common complaints throughout all MS stages with a well-documented negative impact on health-related quality of life [23]. Attributes of the depressive temperament comprise self-denying, low energy-level, negativism, introversion, unhappiness, and always seeing the dark side of things [10]. When facing an unpredictable potentially disabling disease like MS these attributes are certainly not helpful in enhancing health related quality of life. In cyclothymic temperament, rapid shifts in mood and energy levels may predispose to distress and reduced health related quality of life. Surprisingly, statistically significant negative effects of the anxious temperament were only seen in 2 out of 9 MusiQol subscales (i.e., “activities of daily living”, “psychosocial wellbeing”) and not in global index score. Anxiety disorders in their subclinical manifestations may not affect social relationships and other dimensions of health-related quality of life that contribute to MusiQol global index score. According to Akiskal, outstanding characteristics of individuals with hyperthymic temperament are “cheerful, overoptimistic or exuberant, naïve, overconfident, self-assured, boastful, bombastic and grandiose” [10]. This almost over-boarding positive self-assessment may explain the positive impact on overall health-related quality of life and, as this behavior may offend other people, also the negative influence on the MusiQol subscales of “sentimental and sexual life” and “rejection”. MS patients with higher depressive and cyclothymic temperament scores surprisingly reported an increased health related quality of life in the subscales “rejection” and “relationship with health care system”, maybe because they focus more on themselves than on the social environment and therefore don’t experience “rejection”. The positive relationship with the health care system may exist, as these patients might be seen as more vulnerable and needy to doctors, thus, receiving more attention from them. The irritable temperament does not seem to affect health-related quality of life at all, while irritability was found to be a key symptom of interictal dysphoric disorder in epilepsy [24].

Previous studies reporting not on temperament, but on MS patients´ personality characteristics measured various personality traits, used different screening tools, and except for one [25], did not evaluate an association to health-related quality of life, thus, making results barely comparable with ours [5, 6, 11, 12, 26, 27]. Only Zarbo reported an association between personality and health-related quality of life in MS patients, but these results referred to the Five Factor model, underpinning a negative influence of introversion and neuroticism [25]. Moreover, neurotic MS patients were more worried about their future, experienced higher levels of anxiety and depression [25], and were more prone to risky treatment options [28].

Our study has some limitations. First, the number of patients for our final analysis of the MusiQol global index score was lower than expected. However, a sensitivity analysis covering 132 patients supports the validity of our result. Second, we did not collect data from a healthy control group, as we preferred evaluating health-related quality of life with an MS specific questionnaire not applicable for a healthy control group. Another limitation concerns the cross-sectional design of our study and that possibly confounding factors (like cognition and fatigue) were not assessed systematically.

## Conclusion

In conclusion, hyperthymic temperament in MS patients could explain an increased and depressive and cyclothymic temperament a reduced health-related quality of life, independent of current disability status, co-morbid mood-disorders and immunomodulatory treatments. Prospective evaluation of temperament types in MS patients could help to identify patients early, who need more biopsychosocial support to booster quality of life. This includes consecutive initiation of early and sufficient pharmacological as well as non-pharmacological treatment, especially psychotherapy. Evaluating temperaments in MS may also support clinicians to better understand coping strategies, treatment adherence, and decisions on accepting possibly risky treatment options.

## Additional files

Additional file 1: **Table S1.** Linear regression analysis showing the effect of temperament types on MusiQol Global Index Score (sensitivity analysis, *N* = 132). (DOCX 100 kb)

Additional file 2: **Table S2.** Logistic regression analysis showing the effect of temperament types on MusiQol Dimensions 4–9; each dimension dichotomized to ‘full score’ versus ‘below full score’. (DOCX 15 kb)

## Abbreviations

EDSS: Expanded disability status scale
HRQoL: Health related quality of life
MINI: Mini International Neuropsychiatric Interview
MS: Multiple sclerosis
MusiQol: The Multiple Sclerosis International Quality of Life Questionnaire
SDS: Severity of dependence scale
TEMPS-M: Temperament Evaluation of Memphis, Pisa, Paris, and San Diego Questionnaire – Münster version
