# Supplementary material for: Health-related quality of life in multiple sclerosis: temperament outweighs EDSS
Source: BMC Psychiatry. 2018 May 23;18:143. doi: 10.1186/s12888-018-1719-6 (PMC5966924; doi:10.1186/s12888-018-1719-6)
Supplement: Supplementary file 1 — Table S1. Linear regression analysis showing the effect of temperament types on MusiQol Global Index Score (sensitivity analysis, N = 132). (DOCX 100 kb) [file 12888_2018_1719_MOESM1_ESM.docx]

**Supp. Table 2** Logistic regression analysis showing the effect of temperament types on MusiQol Dimensions 4-9; each dimension dichotomized to ‘full score’ versus ‘below full score’.

|  | **N** | **PEV** | **OR (CI)** | **p** | **Adj. p** |  |  |
| --- | --- | --- | --- | --- | --- | --- | --- |
| **Relationships friends** | 114 | 0.113 | - | - | - |  |  |
| Depressive T |  | 0.118 | 1.12 (0.9; 1.40) | 0.286 | 0.573 |  |  |
| Cyclothymic T |  | 0.153 | 1.33 (1.0; 1.74) | 0.038 | 0.153 |  |  |
| Hyperthymic T |  | 0.170 | 0.91 (0.85; 0.99) | 0.023 | 0.116 |  |  |
|  |  |  |  |  |  |  |  |
| Irritative T |  | 0.117 | 1.12 (0.89; 1.4) | 0.334 | 0.573 |  |  |
| Anxious T |  | 0.130 | 1.22 (0.92; 1.62) | 0.170 | 0.511 |  |  |
| **Relationships family** | 121 | 0.059 |  |  |  |  |  |
| Depressive T |  | 0.067 | 1.09 (0.91; 1.30) | 0.367 | 0.854 |  |  |
| Cyclothymic T |  | 0.074 | 1.16 (0.92; 1.45) | 0.214 | 0.854 |  |  |
| Hyperthymic T |  | 0.107 | 0.92 (0.86; 0.98) | 0.015 | 0.076 |  |  |
| Irritative T |  | 0.061 | 0.93 (0.76; 1.15) | 0.508 | 0.854 |  |  |
| Anxious T |  | 0.065 | 0.88 (0.7; 1.10) | 0.257 | 0.854 |  |  |
| **Sentimental/Sexual life** | 130hh | 0.019 |  |  |  |  |  |
| Depressive T |  | 0.061 | 1.22 (1.02; 1.45) | 0.027 | 0.108 |  |  |
| Cyclothymic T |  | 0.044 | 1.21(0.99; 1.49) | 0.068 | 0.205 |  |  |
| Hyperthymic T |  | 0.136 | 0.88 (0.82; 0.94) | <0.001 | 0.001 |  |  |
| IrritativeT |  | 0.030 | 1.11 (0.92; 1.34) | 0.272 | 0.507 |  |  |
| Anxious T |  | 0.027 | 1.13 (0.92; 1.40) | 0.254 | 0.507 |  |  |
| **Coping**  **Depre**  **De**  **Dep** | 120 | 0.052  00000 |  |  |  |  |  |
| Depressive T |  | 0.071 | 1.15 (0.96; 1.39) | 0.128 | 0.511 |  |  |
| Cyclothymic T |  | 0.070 | 1.17 (0.94; 1.46) | 0.164 | 0.511 |  |  |
| Hyperthymic T |  | 0.170 | 0.92 (0.86; 0.98) | 0.015 | 0.075 |  |  |
| Irritative T |  | 0.052 | 1.02 (0.84;1.23) | 0.873 | 0.873 |  |  |
| Anxious T |  | 0.068 | 1.16 (0.92; 1.46) | 0.203 | 0.511 |  |  |
| **Rejection** | 113 | 0.104 |  |  |  |  |  |
| Depressive T |  | 0.240 | 1.5 (1.18; 1.9) | <0.001 | 0.005 |  |  |
| Cyclothymic T |  | 0.172 | 1.42 (1.09; 1.85) | 0.010 | 0.031 |  |  |
| Hyperthymic T |  | 0.198 | 0.9 (0.83; 0.96) | 0.004 | 0.014 |  |  |
| Irritative T |  | 0.129 | 1.23 (0.98; 1.55) | 0.068 | 0.106 |  |  |
| Anxious T |  | 0.142 | 1.3 (1.0; 1.72) | 0.053 | 0.106 |  |  |
| **Rel.Health-care system** | 120 | 0.136 |  |  |  |  |  |
|  |  |  |  |  |  |  |  |
| Depressive T |  | 0.250 | 1.51 (1.21; 1.89) | <0.001 | 0.001 |  |  |
| Cyclothymic T |  | 0.230 | 1.52 (1.18; 1.96) | 0.001 | 0.004 |  |  |
| Hyperthymic T |  | 0.164 | 0.93 (0.87; 0.99) | 0.026 | 0.078 |  |  |
| Irritative T |  | 0.137 | 1.05 (0.86; 1.28) | 0.645 | 0.645 |  |  |
| Anxious T |  | 0.167 | 1.29 (1.01; 1.65) | 0.042 | 0.084 |  |  |
|  |  |  |  |  |  |  |  |
|  |  |  |  |  |  |  |  |
|  |  |  |  |  |  |  |  |

Footnote: PEV: proportion of explained variation. T: temperament, OR: odds ratio quantifying the effect of a 20% increase in the respective temperament score (except hyperthymic temperament: quantifying the effect of a unit increase in the temperament score); CI: 95% confidence interval. Adjusted p: adjusted p-value for testing five temperaments
