# Supplementary material for: Health-related quality of life in multiple sclerosis: temperament outweighs EDSS
Source: BMC Psychiatry. 2018 May 23;18:143. doi: 10.1186/s12888-018-1719-6 (PMC5966924; doi:10.1186/s12888-018-1719-6)
Supplement: Supplementary file 2 — Table S2. Logistic regression analysis showing the effect of temperament types on MusiQol Dimensions 4–9; each dimension dichotomized to ‘full score’ versus ‘below full score’. (DOCX 15 kb) [file 12888_2018_1719_MOESM2_ESM.docx]

**Supp. Table 1.** Linear regression analysis showing the effect of temperament types on MusiQol Global Index Score (sensitivity analysis, N=132).

|  | **R^2^** | **effect (CI)** | **p-value** | **Adj. p-value** |  |  |
| --- | --- | --- | --- | --- | --- | --- |
| Global Index Score | 0.322 |  | - | - |  |  |
| Depressive T | 0.395 | -1.9 (-2.9; -0.9) | <0.001 | 0.001 |  |  |
| Cyclothymic T | 0.460 | -3.2 (-4.3;-2.1) | <0.001 | <0.001 |  |  |
| Hyperthymic T | 0.426 | 0.8 (0.5;1.1) | <0.001 | <0.001 |  |  |
|  |  |  |  |  |  |  |
| Irritative T | 0.323 | -0.2 (-1.4;0.9) | 0.668 | 1.0 |  |  |
| Anxious T | 0.350 | -1.5 (-2.8;-0.2) | 0.022 | 0.088 |  |  |

**Footnote:** effect: regression coefficient quantifying the effect of a 20% increase in the respective temperament score (except hyperthymic temperament: quantifying the effect of a unit increase in the temperament score); CI: 95 % confidence interval; adj. p-value: adjusted for testing five temperaments (Bonferroni-Holm method). T: Temperament.
